# Inverted T-shape free gingival graft for root coverage in advanced recession of mandibular incisors: A case series

**DOI:** 10.6026/973206300213114

**Published:** 2025-09-30

**Authors:** Sangita Agarwal, Neha Sahu, Sanjum Aasma, Supriya Mishra, Alka Snehil

**Affiliations:** 1 Department of Periodontology, Government Dental College, Raipur, India

**Keywords:** Diastema-associated recession, inverted T-shaped FGG, Root coverage, soft tissue augmentation

## Abstract

Extensive gingival recession in the mandibular anterior region with diastema poses both functional and aesthetic challenges, where conventional free gingival grafts (FGG) often fail to provide adequate results in cases of combined vertical and horizontal tissue deficiencies. Hencce, an inverted T-shaped FGG design has been introduced, enabling simultaneous root coverage and interdental tissue augmentation. The technique involves a vertical graft segment to address recession depth and a horizontal arm to bridge the diastema, thereby enhancing stability, vascularization, and esthetic harmony. In this case series, all patients showed favorable outcomes with significant root coverage, increased width of keratinized tissue, and improved gingival contours. These results highlight the inverted T-shaped FGG as a predictable and esthetically satisfying option for managing complex recession defects complicated by diastema.

## Background:

A free gingival graft (FGG) is considered the gold standard for increasing the width and thickness of keratinized tissue. It also treats gingival recession and increases vestibular depth, but it has limitations such as color mismatch, donor site morbidity, and incomplete root coverage in advanced recession cases [1, 2]. Root coverage in recession type 2 and 3 (RT2 and RT3) defects is particularly challenging and often unpredictable due to severe interdental bone and soft tissue loss [3]. The literature provides only limited techniques for treating RT2 and RT3 cases to achieve root coverage. Reported approaches include connective tissue grafts (CTGs), free gingival grafts (FGGs) [4], and the connective tissue graft wall technique [5], with and without modifications. Several modifications have been introduced to FGGs to enhance predictability, such as gingival unit grafts (GUGs) [6] and partially epithelized FGGs [7]. Vertical recession reduction and keratinized tissue gain were reported to be significantly higher in the GUG group compared to conventional FGGs [8]. Additionally, utilizing interdental gingival tissues may improve graft survival by benefiting from better blood perfusion of the recipient site [9]. Partial root coverage and papillary reconstruction were demonstrated with the inverted T-shaped FGG, introduced by Alrmali *et al.* in 2022 [10]. Therefore, it is of interest to describe three additional cases in which the inverted T-shaped FGG was used to achieve root coverage and interdental tissue improvement in RT2 defects.

## Case 1:

A 38-year-old female patient reported to Department of Periodontics, Government Dental College and Hospital, Raipur, with the chief complaint of heavy deposits on the lower front teeth and noticeable flaring of the mandibular anterior teeth, which developed over a short duration of approximately one month. The patient reported no history of any systemic illness and was free from any known deleterious habits such as smoking or drug use. Oral hygiene practices were found to be inadequate, contributing to the clinical condition. On clinical examination, two distinct areas of gingival recession were noted in the mandibular anterior teeth. The recession measured approximately 3 mm in width by 3 mm in depth on 31, and 3 mm in width by 2mm in depth on 41, with the presence of a thin gingival biotype, blunt interdental papilla and decreased keratinized tissue width (Table 1 - see PDF). The tension test was positive, indicating the involvement of a high and prominent mandibular labial frenum leading to poor plaque control. No evidence of trauma from occlusion with involved teeth was observed but teeth were grade I mobile. Horizontal bone loss extending to the middle third of the root was observed in intra-oral periapical radiograph. Based on clinical and radiographic findings a diagnosis of localized stage III grade B was given with RT2 recession. Written informed consent was obtained once the treatment plan was explained. A palatal splint was constructed prior to surgery. The recipient site was prepared by making a horizontal incision at the mucogingival junction (MGJ) as well as two vertical incisions extending to the adjacent teeth and about 3-4 mm beyond the MGJ. The surfaces between these incisions were de-epithelialized including interdental papilla using a 15c blade and microsurgical scissor. The exposed root surface was planned with hand instruments and rinsed with saline only. The palatal donor site was designed 2 mm away from the gingival margin using the template in the form of an inverted T-shape. Care was taken to obtain an even thickness of 1-1.5 mm. The final graft height was about 12 mm with a 3 mm extension for interproximal papilla and width was 6mm. This was a case with full dentition; the 3 mm extension of the harvest was extended anteriorly. Next, the graft was contoured, adapted, and sutured at the level of the base of the interdental papilla. The sutures were removed after 2 weeks. For the first 3weeks, the patient was advised not to brush at the surgical site, avoid hard food, and rinse twice daily with 0.2% chlorhexidine digluconate mouthwash. Afterwards patient was asked to resume gentle brushing using a soft toothbrush. Patient was recalled every week during the first month and then every 3 months post-surgery.

## Case 2:

A 23-year-old female patient presented with a chief complaint of spacing between lower front teeth, which developed over approximately two years. Medical history reported was not relevant and dental history revealed difficulty with tooth brushing in that area leading to compromised oral hygiene. On clinical examination, gingival recession was noted with mandibular anterior teeth, specifically presenting as RT2 defects with 31 and RT1 with 41. The recession measured approximately 3 mm in width by 5 mm in depth with grade I Mobility in 31 and 2.5mm in width by 1 mm in depth with 41, with the presence of thin gingival biotype. The tension test was positive, indicating the involvement of a high and prominent mandibular labial frenum and inadequate attached gingiva with 31 (Table 1 - see PDF). Trauma from occlusion is also found with upper and lower incisors). On radiographic examination bone loss was up to cervical third of root of 31 and 41. A diagnosis of localized periodontitis stage II grade B with 31 and 41 was made. Following completion of phase I therapy a reduction in tooth mobility was observed. This case was also managed surgically with t shape free gingival graft similar to case 1.

## Case 3:

A 30-year-old female patient, presented with the chief complaint of flaring of her upper and lower anterior teeth, worsened with time. She also noticed the presence of visible deposits on her teeth. The patient gave no relevant systemic history and denied any deleterious habits, such as smoking or the use of tobacco products. Her oral hygiene was found to be inadequate upon examination. Initial clinical evaluation revealed generalized gingival inflammation with bleeding on probing, consistent with a diagnosis of generalized gingivitis dental biofilm-induced. A heavy ring of subgingival calculus was noted in the lower anterior region, extending up to the mucogingival junction. There was no evidence of trauma from occlusion. Full-mouth scaling and root planing (SRP) was carried out, and the patient was instructed for proper oral hygiene practices. She was scheduled for a follow-up after 15 days. At the 15-day follow-up, re-evaluation showed marked improvement in overall gingival inflammation. However, teeth 31 and 41 exhibited Cairo's RT2 type gingival recession, associated with a midline diastema and Grade I tooth mobility. Tooth 41 demonstrated a recession depth of 5 mm with 3 mm of recession width, while tooth 31 showed a 3mm recession depth with 3 mm of recession width (Table 1 - see PDF). The gingival tissue thickness in the area was measured to be approximately 1.8 mm. Radiographic evaluation revealed horizontal bone loss extending to the coronal third of the root surfaces in the affected area. Based on the clinical and radiographic findings, a revised diagnosis of localized periodontitis Stage II, Grade B with 31 and 41 was made. This case was also surgically managed with inverted T- shaped free gingival graft.

## Discussion:

The free gingival graft (FGG) continues to be a dependable technique for augmenting keratinized tissue in regions where it is deficient or absent [1, 12]. Allen and Cohen later advanced this concept by introducing the gingival unit graft (GUG), designed with extended dimensions to enhance graft survival [9]. Comparative studies have shown that GUG provides greater improvements in recession reduction, attachment gain, and keratinized tissue width (KTW) compared to conventional FGG [8]. In a randomized controlled trial by Sriwil *et al.* (2020), a modified GUG demonstrated superior outcomes in treating Miller Class III recession defects over the traditional FGG [11]. In the present case series, the use of a T-shaped FGG showed favorable results, including enhanced interproximal tissue quality, gingival thickness, partial root coverage, and increased KTW (Table 1 - see PDF). The improved predictability of this approach may be attributed to the increased vascularized interproximal bed, which enhances graft survival. A key consideration for the success of this technique is the presence of adequate interproximal space, enabling extension of the graft to the lingual gingiva and secure suturing.

## Conclusion:

The T-shaped free gingival graft represents a reliable modification of the conventional technique for managing advanced recession defects associated with diastema. By improving graft stability, vascularization, and tissue integration, it provides predictable root coverage, increased keratinized tissue width, and better esthetic outcomes. This technique can be considered a valuable and esthetically satisfying option in complex clinical situations where both vertical and horizontal soft tissue deficiencies coexist.

## Source of Support and Funding:

Self-Funded.

Additionally, we noted better improvement in vertical recession when followed for 6 months, due to the creeping attachment phenomenon. We can conclude that T shaped free gingival graft can lead to partial root coverage, increased keratinized gingival tissue width and thickness even in advanced recession cases. However, randomized clinical trials are needed to confirm this enhanced clinical outcome.
